# Monte Carlo studies of two-dimensional polymer–solvent systems

**DOI:** 10.1007/s00894-017-3216-0

**Published:** 2017-02-09

**Authors:** Piotr Polanowski, Jeremiasz K. Jeszka, Andrzej Sikorski

**Affiliations:** 1 Department of Molecular Physics, Technical University of Łódź, 90-924 Łódź, Poland; 2 Department of Man-Made Fibres, Technical University of Łódź, 90-924 Łódź, Poland; 3 Department of Chemistry, University of Warsaw, Pasteura 1, 02-093 Warsaw, Poland

**Keywords:** Cooperative motion algorithm, Monte Carlo simulations, Polymer melts, Static properties, Thin films

## Abstract

The static properties of two-dimensional athermal polymer solutions were studied by performing Monte Carlo lattice simulations using the cooperative motion algorithm (CMA) and taking into account the presence of explicit solvent molecules. The simulations were performed for a wide range of polymer chain lengths *N* (16–1024) and concentrations *φ* (0.0156–1). The results obtained for short chains (*N* < 256) were in good agreement with those given by previous simulations. For the longest chains (512 or 1024 beads), some unexpected behavior was observed in the dilute and semidilute regimes. A pronounced change in the concentration dependence of chain size and shape was observed below a certain critical concentration (0.6 for the longest chains under consideration, consisting of 1024 beads). Longer chains became more extended below this concentration. The behavior of the single-chain structure factor confirmed these changes in the fractal dimension of the chain as a function of the concentration. The observed phenomena are related to the excluded volume of solvent molecules, which causes the chain statistics to be modified in the vicinity of other chains; this effect is important in strictly 2D systems.

**Graphical abstract:**
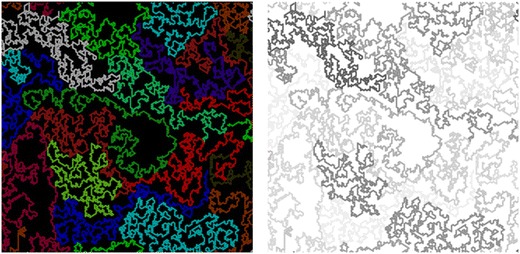
Extended long chains at moderate density with solvent molecules inside coils.

Extended long chains at moderate density with solvent molecules inside coils.

## Introduction

The behavior of polymer chains in two-dimensional systems has attracted considerable interest in recent years [1–19]. Elucidating this behavior is important for understanding the properties of macromolecules that are strongly adsorbed on surfaces, including biological systems. A two-dimensional system containing polymer chains can also be considered a limiting case of systems consisting of a polymer intercalated in layered silicates. The investigation of polymer ultrathin films has recently become one of the most interesting research directions in materials science. This is due to the enormous success of organic electronic devices such as OTFTs (organic thin-film transistors), OPVDs (organic photovoltaic devices), and OLEDs (organic light-emitting diodes), which offer unique advantages over well-known amorphous silicon electronics [20]. These advantages include high throughput, inexpensive production, mechanical flexibility, light weight, efficient integration with electronic circuits, and low power consumption. The above advantages mean that technology based on ultrathin organic films is very promising, although the speed of organic electronic devices is not that high. On the other hand, the case of a two-dimensional athermal polymer solution is very interesting from a polymer physics perspective. This is because strong excluded-volume interactions lead to behavior that is not observed in the three-dimensional case. Moreover, two-dimensional systems, treated for many years in polymer physics as strictly theoretical, have since been obtained practically and studied in a series of experiments. Maier and Rädler used labeled DNA molecules adsorbed on the surfaces of charged lipid bilayers [3, 4], Lin at al. studied labeled DNA conformations in nanoslits [11], and Aoki et al. used near-field optical microscopy to study ultrathin layers of perylene-labeled poly(isobutyl methacrylate) prepared via Langmuir–Blodgett or spin-coating techniques [8, 9]. These experiments in which conformations of single macromolecules were observed directly show that, in this situation, DNA molecules exhibit two-dimensional conformational behavior, in good agreement with theoretical predictions. The polymer escape transition of tethered chains in two dimensions has also been studied [10]. Granular chains confined in a two-dimensional glass container were investigated experimentally and found to show Gaussian behavior at high densities [13]. AFM studies of quasi-2D and real 2D polymers [18, 19] highlighted structural differences between single chains and dense systems, as the single chains exhibited typical Gaussian behavior while the dense system involved strong mutual interpenetration.

The behavior of long chains in 2D systems has been a rather controversial topic in the literature. De Gennes suggested that, as chain interpentration was not possible in 2D, the chains should adopt a disc-like conformation at high concentrations, with other chains being practically excluded from the region on the surface occupied by a given coil [21]. However, computer simulations did not confirm this effect, although it should be noted that the simulated chains were not very long, ranging from 100 [22] to 256 [1, 7] beads. Meyer et al. observed non-Gaussian behavior of a long chain in a dense 2D polymer system using molecular dynamics simulation [14]. This kind of behavior was also seen in other experiments concerning dense polymer 2D systems [18], but it was not confirmed by the direct observation of granular 2D chains [13]. On the other hand, Vlahos and Kosmas [2] analyzed the effects of interaction parameters and chain length on phase diagrams of polymer mixtures using the Edwards-type Hamiltonian. The results obtained by those authors indicate that phase separation is possible in mixtures of chemically identical linear homopolymers of different sizes within specific ranges of chain disparity and concentration. This should also result in significant changes in the conformational properties of the chains. Studies of chemically identical homopolymer blends were also carried out for the 3D bulk case; it was shown that stability can move in either direction when shifting from a 2D to a 3D system [23]. These studies were extended by performing Monte Carlo simulations of the influence of the chain architecture on the miscibility of the polymer blend [24, 25]. Experiments have also provided detailed information on chain conformations in a dilute solution [26, 27] and in dense systems containing long chains [13], but the polymer concentration region from 0.1 to 0.9 has not been explored experimentally in depth except in relation to the percolation problem associated with polymer chains in 2D [28–32] and dynamics [33]. Thus, computer simulation is the method of choice for such polymer concentrations in 2D. Such studies have been performed using various methods: the reptation method [22], self-avoiding random walk (SAW) [5], Brownian dynamics [34], the bond fluctuation model [35], off-lattice MC simulations [1], and molecular dynamics [36], but the ranges of chain length and polymer concentration investigated have not been wide enough to unambiguously exclude or confirm certain effects.

In our previous paper [37], we reported the results obtained from Monte Carlo simulations of 2D athermal polymer solutions performed using the cooperative motion algorithm (CMA) developed by T. Pakula [38–41]. This algorithm enables simulations of dense systems to be realized, and is efficient enough to allow long-chain systems (up to 1024 beads in one chain in this case) to be studied. We showed that the behavior of concentrated solutions of long-chain polymers is qualitatively different from that of shorter-chain polymers. Moreover, for the longest chains considered (consisting of 512 and 1024 segments), a kind of microphase transition was observed (domains of pure solvent of a similar size to the chains themselves appeared in those systems). In the present paper, we summarize the results of a detailed analysis of the influence of concentration on polymer chain size and structure, with the full range of polymer concentrations considered.

## Methods

In simulations employing the cooperative motion algorithm (CMA), ensembles of beads located at lattice sites are connected by unbreakable bonds to form structures representing macromolecules [38–45]. All lattice sites are occupied, so this model represents dense systems such as polymer melts. The results presented here were obtained by performing simulations on a 2D triangular lattice. The coordination number of the lattice was six, i.e., each monomer had six nearest neighbors. The bond length was equal to 1.

Each lattice site could only be occupied by a single molecular element (a polymer bead or a solvent particle), i.e., the excluded-volume condition was applied to the system. In such systems, strictly cooperative dynamics involving rearrangements that satisfy the local continuity condition are employed (no empty lattice sites are generated). A segment of one chain can move only if neighboring segments of the same chain, segments of different chains, or solvent molecules move simultaneously. This is realized by introducing local motions consisting of displacements of a certain number of molecular elements along closed loops, meaning that each element replaces one of its neighbors in such a way that the sum of displacements of the elements taking part in the rearrangement is zero (continuity condition). During such rearrangements, the model macromolecules undergo conformational transformations while preserving their identities. All conformations that satisfy these conditions are allowed. In this model, the relative probabilities of conformations do not have to be modified a priori. If the available conformations of a chain are restricted by the presence of impenetrable walls or parts of other chains, adjustments occur based on the feasibility of closing the displacement loops involving this conformation. This is why the CMA model has been successfully used to simulate complex macromolecules such as multiarm stars [24] and linear chains in a confined space [24, 25].

Quantities characterizing the system were calculated between cooperative rearrangement steps. The time step corresponds to the number of simulation steps that must occur before an average of one attempt to move each bead has been made. Although the CMA algorithm has not been rigorously shown to be ergodic for any polymer system, it has been proven to be ergodic for dimers by Reiter [38–40]. The need for a detailed balance in the athermal polymer system considered here corresponds to showing that the transition probabilities between two neighboring states are equal. In this algorithm, two such states are always reversible and are separated by cooperative rearrangements along cooperative loops of the same size and form but with different directions of motion. Because a loop consists of vectors that can point in any direction with equal probability, this condition is satisfied. Moreover, it remains valid for any polymer system because the loops are independent of the structure. More details about the algorithm used are given elsewhere [38–41]. We considered a two-dimensional system of flexible polymer chains immersed in a solvent (in the case of a polymer melt, the system was filled with polymer beads only). The size of a solvent molecule was the same as that of the monomer. The model used here consisted of 256 × 256 beads, i.e., its edge was longer than the average end-to-end distance of the longest simulated chain (1024 beads). Periodic boundary conditions were employed in all directions. To be sure that the effects observed for the longest chains (1024 beads) did not arise because the simulated system was too small (especially at low polymer concentrations), we also performed simulations of these chains in a larger system of 512 × 512 beads.

The polymer concentration *φ* is defined as the ratio of the sites occupied by the polymer beads to the total number of lattice sites. Thus, *φ* = 1 means that all the sites are occupied by polymer beads. The condition in which we must have an integer number of chains in the simulation box imposes restrictions on the concentrations studied. For instance, when *N =* 1024 and one chain is present in the box, *φ* = 1024/256^2^ = 0.015625; for two chains,* φ *= 0.03125, etc. It should be noted that this definition is different from that used in off-lattice simulations. For instance, Yethiraj [1] defines the concentration as the ratio of the sum of the surface areas covered by the circles representing polymer monomers to the total surface area. This means that the maximum concentration available corresponds to the close packing of circles, so it is equal to ca. 0.9069, and therefore a related correction should be made when comparing our results with Yethiraj’s.

At the beginning of the simulations, the polymer chains were fully extended in the *x* direction (and folded if necessary). The equilibration of the system was monitored by observing several parameters. It was observed that all of the monitored quantities (defined in the next section) reached their equilibrium values after approximately the same simulation time had elapsed. An example of this time dependence is shown in [37] for *R*_g_. The equilibrated systems obtained in this way were used as input systems in the simulations discussed below.

## Results and discussion

### Parameters determined

The conformational properties of the chains in the simulations were monitored by calculating the following parameters:

- Mean square radius of gyration 〈*R*_g_^2^〉:
  1 $$ \left\langle {R}_{\mathrm{g}}^2\right\rangle =\left\langle \frac{1}{N}{{\displaystyle \sum_{i=1}^N\left({\mathbf{r}}_i-{\mathbf{r}}_{\mathrm{cm}}\right)}}^2\right\rangle, $$
  where *N* is the total number of beads constituting the chain and ***r***_*c*m_ is the coordinate of the center of mass of the chain.
- Gyration tensor *T*:
  2 $$ {T}_{kl}=\left\langle \frac{1}{N}{\displaystyle \sum_{i=1}^N\left({r}_{i k}-{r}_{\mathrm{cm}, k}\right)\left({r}_{i l}-{r}_{\mathrm{cm}, l}\right)}\right\rangle, $$
  where *k* and *l* are the coordinates *x* and *y*, *r*_*ik*_ is the *k*th coordinate of position ***r***_*i*_, and *r*_cm,*k*_ is the *k*th coordinate of the center of mass of the chain. The tensor *T* has two eigenvalues—denoted *λ*_1_ and *λ*_2_ (with the convention *λ*_1_ ≤  *λ*_2_)—which fulfill the relation
  3 $$ {R}_{\mathrm{g}}^2={\lambda}_1+{\lambda}_2. $$
- Asphericity parameter *A*_2_, defined as
  4 $$ {A}_2=\frac{\left\langle {\left({\lambda}_2-{\lambda}_1\right)}^2\right\rangle }{\left\langle {\left({\lambda}_2+{\lambda}_1\right)}^2\right\rangle }, $$
  which means that *A*_2_ = 1 for a fully extended chain and *A*_2_ = 0 for a disk.
- Intramolecular site–site correlation function for sites separated by *r* = |**r**_*i*_ − **r**_*j*_|:
  5 $$ \gamma (r)=\frac{1}{N}\left\langle c\left({\mathbf{r}}_i\right)\cdot c\left({\mathbf{r}}_j\right)\right\rangle, $$
  where *c* is a contrast operator that takes a value of 1 for sites occupied by elements of the same chain and a value of 0 for every other site.
- Static structure factor:
  6 $$ S(q)={\displaystyle \sum_{ij}\gamma (r)}\frac{ \sin \left( q r\right)}{qr}, $$
  where *q* is the scattering vector and* γ* denotes the bead-to-bead correlation function (Eq. 5).
- Pair center-of-mass correlation function, which characterizes chain packing and was calculated using the following definition:
  7 $$ {g}_{\mathrm{cm}\_\mathrm{cm}}(r)=\frac{1}{\phi^2}\left\langle {\displaystyle \sum_i^n{\displaystyle \sum_j^n\delta \left({r}_i\right)\delta \left({r}_j- r\right)}}\right\rangle, $$
  where *r*_*j*_ denotes the position of the center of mass of the *j*th chain.

### Chain size

**Fig. 1:**
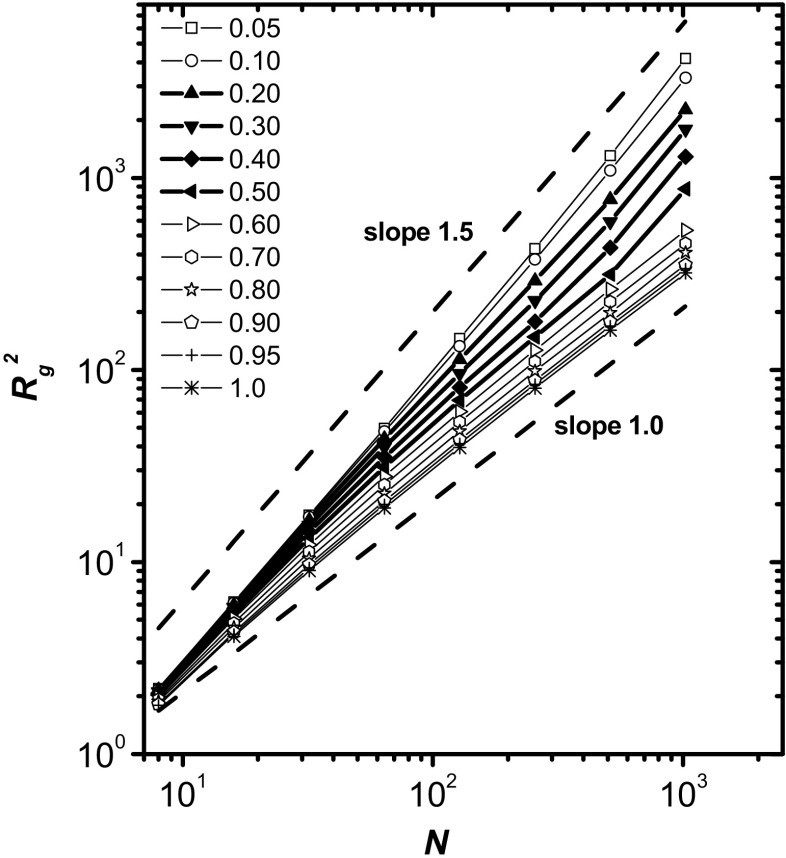
Chain-length dependence of the mean square radius of gyration *R*_g_^2^ for various polymer concentrations (*φ* = 0.05, …, 1.0). The *thicker lines *correspond to *φ* = 0.2, 0.3, 0.4, and 0.5

Figure 1 shows the chain-length dependence of the mean square radius of gyration *R*_g_^2^ for various polymer concentrations *φ*. A very similar picture was obtained for the mean-square end-to-end distance *R*_ee_^2^. In principle, these quantities should scale with the chain length as [21, 46, 47]

8 $$ \left\langle {R}_{\mathrm{g}}^2\right\rangle \propto {N}^{2\nu}. $$

In 2D systems, 2*ν* should be equal to 1 for a single ideal chain or for a chain in a melt, and equal to 1.5 for dilute solutions in good solvent conditions. (The exponent 2*ν* was determined experimentally for single chains and found to be 1.30 in a quasi-2D system [11] and 1.58 in 2D systems [3]). Therefore, in a good solvent, when shifting from a dilute regime to a dense polymer melt, it is expected that the exponent 2*ν* will change from 1.5 to 1. In Fig. 1, we can see that this relationship is generally fulfilled, but there are some significant deviations from it. The curves for *φ* values of between 0.2 and 0.5 (thicker lines) have an s-like shape. The slope of the curve is ca. 1.25 for short polymers, but above *N* = 64 the slope decreases, becoming equal to 1 between *N* = 128 and 256. 2*ν* > 1 is observed because the solutions containing short chains are at semidilute concentrations while the solutions containing long chains are concentrated solutions [29]. This effect is related to the concentration-dependent correlation length of the chain in the semidilute regime, which can also be described using the concept of blobs (see below) [21]. What is surprising is that the exponent 2*ν* increases again for the longest chains (*N* > 256), slightly exceeding the theoretical value of 1.5. This increase over the intermediate concentration range may be explained by the microphase separation effect [36]. However, this effect is also clearly seen for the lowest polymer concentration studied (*φ* = 0.05). It seems, therefore, that it must be related to strong excluded-volume interactions in 2D systems [3], especially when solvent molecules are explicitly taken into account. It appears that chains tend to be more rod-like in such polymer–solvent systems (the reasons for this behavior will be discussed in subsequent sections).

**Fig. 2:**
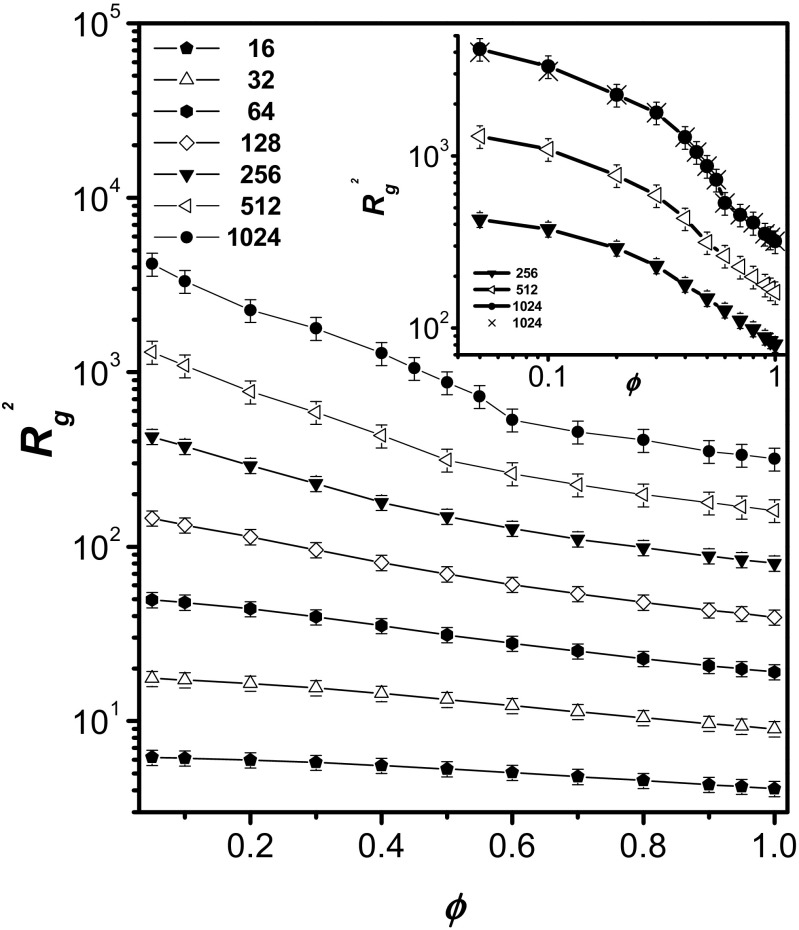
Concentration dependence of the mean square radius of gyration *R*_g_^2^ for various chain lengths. The* error bars* are also shown for all chain lengths. The* inset* shows the results for the longest chains (*N* = 256, 512, and 1024) plotted in double-logarithmic coordinates. The values of *R*_g_^2^ for the longest chains (*N* = 1024) in a large box (512 × 512) are indicated by* crosses*

**Fig. 3:**
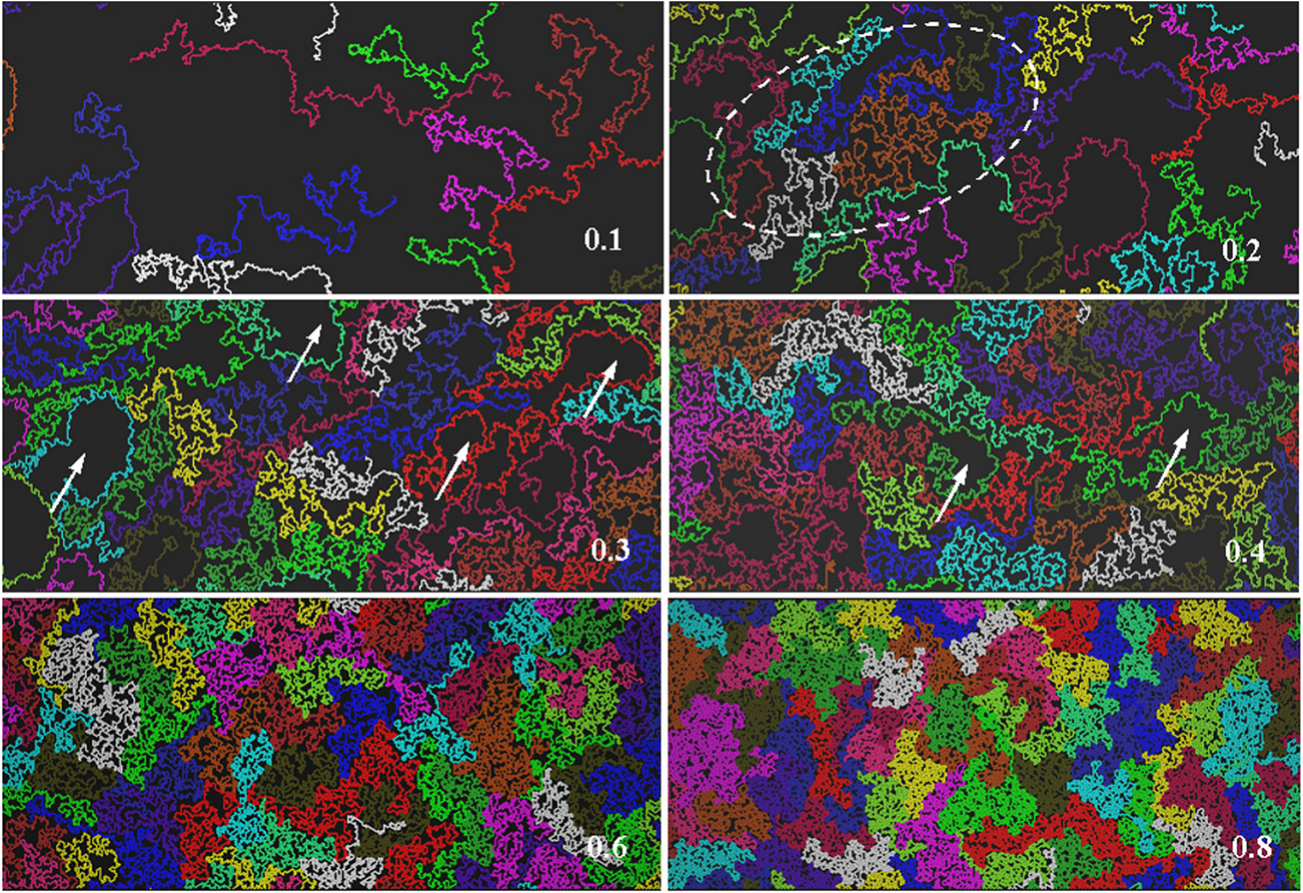
Snapshots of simulated systems for* N* = 512 at various polymer concentrations. A region of high polymer concentration for *φ* = 0.2 is denoted by a* dashed ellipse*. The* arrows in the central panels* indicate larger domains of pure solvent in the phase-separated systems (for *φ* = 0.3 , 0.4)

Figure 2 shows the mean square radius of gyration *R*_g_^2^ as a function of the polymer concentration *φ* for various chain lengths. To better illustrate the conformational changes involved, the results for the longer chains (*N* = 256, 512, and 1024) are presented in double-logarithmic coordinates in the inset. It can be seen that in all cases, *R*_g_^2^ decreases with increasing polymer concentration, as predicted by various theories [21, 46]. The following scaling prediction has been suggested:

9 $$ \left\langle {R}_{\mathrm{g}}^2\right\rangle \propto {\varphi}^{\left(1-2\nu \right)/\left( d\nu -1\right)}, $$

where *d* is the spatial dimension of the system. In the 2D case, we find that *R*_g_^2^ ∼ *ϕ* ^−1^. This scaling behavior is valid for high concentrations, whereas the concentration dependence levels off for dilute systems. It is apparent that, for the longest chains, there is a transition between the semidilute regime and the concentrated regime, which we have interpreted [37] as being due to a microphase separation. This transition is clearly visible in spite of the fact that the errors in the values of *R*_g_^2^ for longer chains are considerably larger than those for shorter chains. When we refer to a microphase separation, we mean the temporary formation of large solvent bubbles inside the chain contour. These domains of solvent disappear during the simulation but others are being formed. This phenomenon takes place in the *φ* range between 0.6 and 0.2 for *N* = 1024 and between 0.5 and 0.3 for *N* = 512. It is worth noting that the chain size increases considerably in the microphase separation region and is not constant as might be expected. This increase is related to the fact that the microdomains of the solvent are surrounded by parts of some of the chains that must be stretched (as can be observed in Fig. 3, where snapshots of the systems studied are presented). The inset in Fig. 2 presents results for longer chains on a log-log scale. The two different regimes of the chain’s size behavior are clearly visible in this figure. In order to check if the size of the simulation box influences the structures of longer-chain systems at densities where microphase separation appears to occur, we performed additional simulations with the length of the Monte Carlo box edge doubled (512 × 512). These results can also be viewed in the inset in Fig. 2. The chain size in the larger box differs from that in the smaller box only at very low concentrations, and the differences are rather small. However, the shape of the *R*_g_^2^(*ϕ*) curve does not change, so the size of the Monte Carlo box cannot be responsible for the presence of two regimes.

**Fig. 4:**
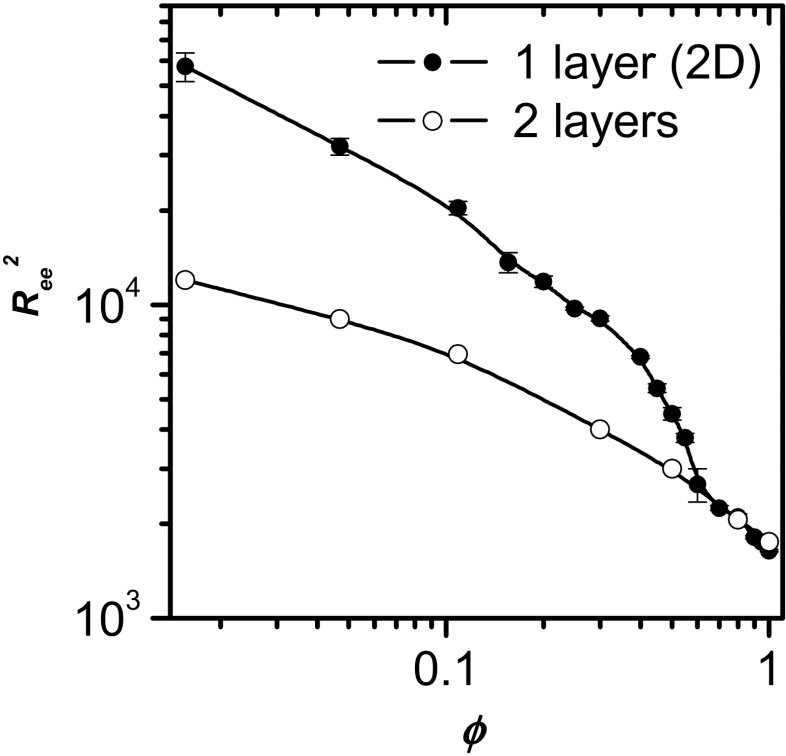
A comparison between 2D and quasi-2D (two-layer) simulations for the chain *N* = 1024 in terms of the concentration dependence of *R*_ee_^2^

To support the supposition that the difficulty encountered by solvent molecules when attempting to pass over the top of a long chain is an important problem in a strictly 2D system with long chains, we also performed some additional simulations for a quasi-2D system. In this case, the simulation box contained not one but two layers of polymer beads. For quite long chains, adding a second layer should not change the chain size and shape—the third eigenvalue of the gyration tensor is negligible in this case, e.g., for *N* = 1024 at *φ* = 0.8, this eigenvalue (*λ*_3_) is less than 0.2% of *λ*_1_. Figure 4 shows a comparison between the 2D and quasi-2D simulations performed for the chain *N* = 1024 in terms of the concentration dependence of the mean-square end-to-end distance *R*_ee_^2^. It is clear that the curves split below the density (*φ* = 0.6) at which the microphase separation was suggested to occur: the size of the strictly 2-D chain increases and the difference between the curves becomes quite pronounced at low polymer concentrations. This anomalous increase in the radius of gyration is not observed in the two-layer system. The supposed microphase separation (the formation of solvent bubbles inside the chain contour) does not appear here, as solvent molecules can easily move in a relatively short time from one side of the chain to the other via the second layer.

### Chain shape and packing

**Fig. 5:**
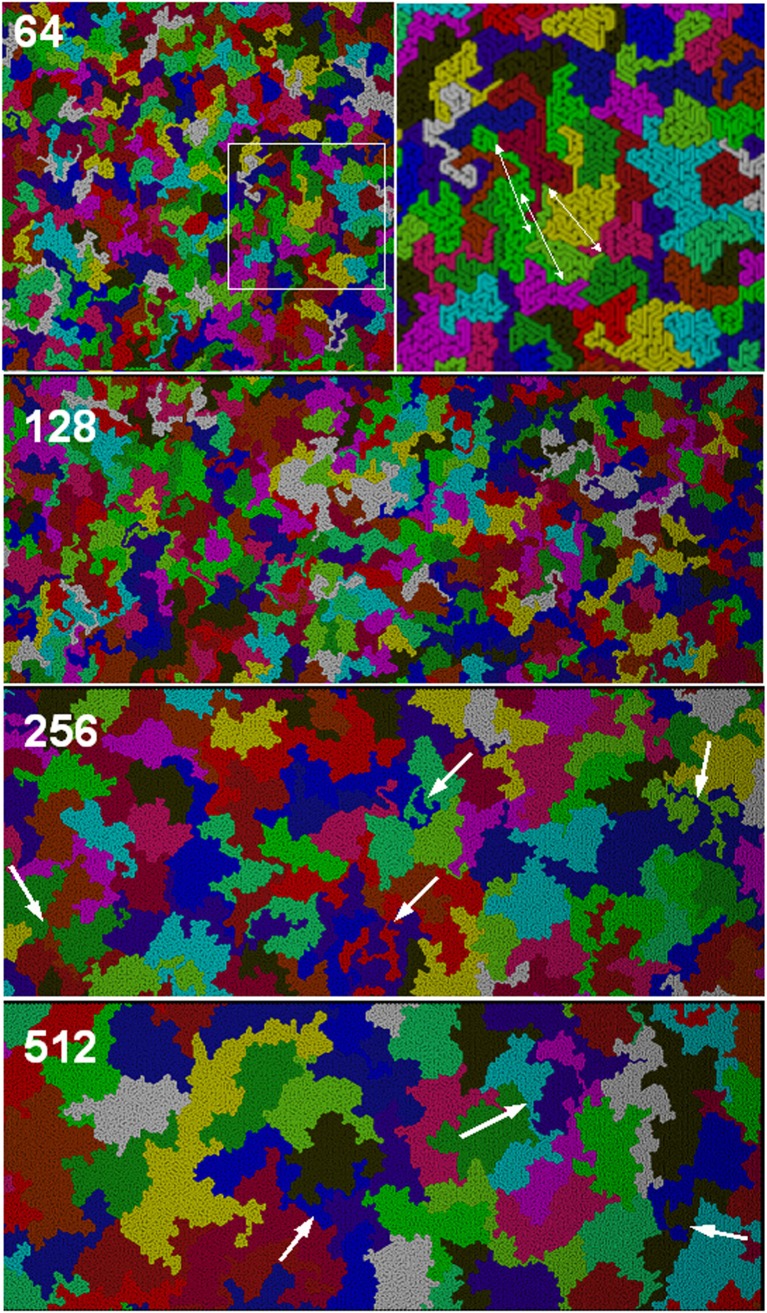
Snapshots of simulated systems at full polymer occupancy *(φ* = 1.0) for selected chain lengths. The* top right panel* shows an enlargement of the* left-hand panel* (the enlarged area is indicated by the* square*). For *N* = 64, the* arrows *indicate the ends of three green chains. The* arrows* for systems *N* = 256 and 512 indicate chains with exotic shapes, which are generated when chains interpenetrate other coils

Figure 5 shows typical snapshots of polymer melts (*φ* = 1) for various chain lengths. It can be seen that some chains adopt a compact, disc-like form, with other chains completely excluded from the coil area (as suggested originally by de Gennes [21]), while other chains can be found in extended configurations or have a dumbbell shape with two compact domains joined by a stretched fragment. Many long chains even penetrate deeply into the discs of other coils, resulting in exotic forms such as those indicated by arrows in Fig. 5. The presence of disc-like chains and of interpenetrated discs was observed for both short and long chains. Shorter chains (also shown in the zoomed snapshot for *N* = 64 in Fig. 5) have various shapes but coil interpenetration is rare. Please note that although all of the chains in the same snapshot are the same length, neighboring chains sometimes have practically the same color, so two or even three chains look like one very large coil (this effect is highlighted for three green chains indicated by arrows in the top right panel of Fig. 5, which shows a zoomed area of the top left panel).

Snapshots of the simulated systems for *N* = 512 at different polymer concentrations are presented in Fig. 3. It is clear that there is a big difference in chain conformation between the concentrated and diluted regimes. In concentrated solutions, most of the chains adopt a compact conformation similar to that seen in the melt (see Fig. 5) in which the surface area occupied by the coil is densely filled by the coil. Thus, the coil size is small. On the contrary, in the diluted regime, the chains are mostly extended, so the average coil size is much larger. The picture for *φ* = 0.1 closely resembles fluorescent microscopy images of adsorbed DNA chains presented in [3, 4]. The coil contraction that occurs with decreasing dilution is much more pronounced in the 2D than in the 3D case due to strong excluded-volume interactions—the chains cannot cross each other, so they are much more compact in the melt. In the concentration range 0.3–0.4, relatively large domains of pure solvent appear; this phase separation is discussed in more detail in [37]. Such domains are usually largely surrounded by part of a chain, which acts as a domain border. Also, for* φ* = 0.2, the system is not quite homogeneous; regions with high polymer concentrations (marked by an ellipse in Fig. 3) are separated by domains in which the polymer concentration is significantly lower.

**Fig. 6a–b:**
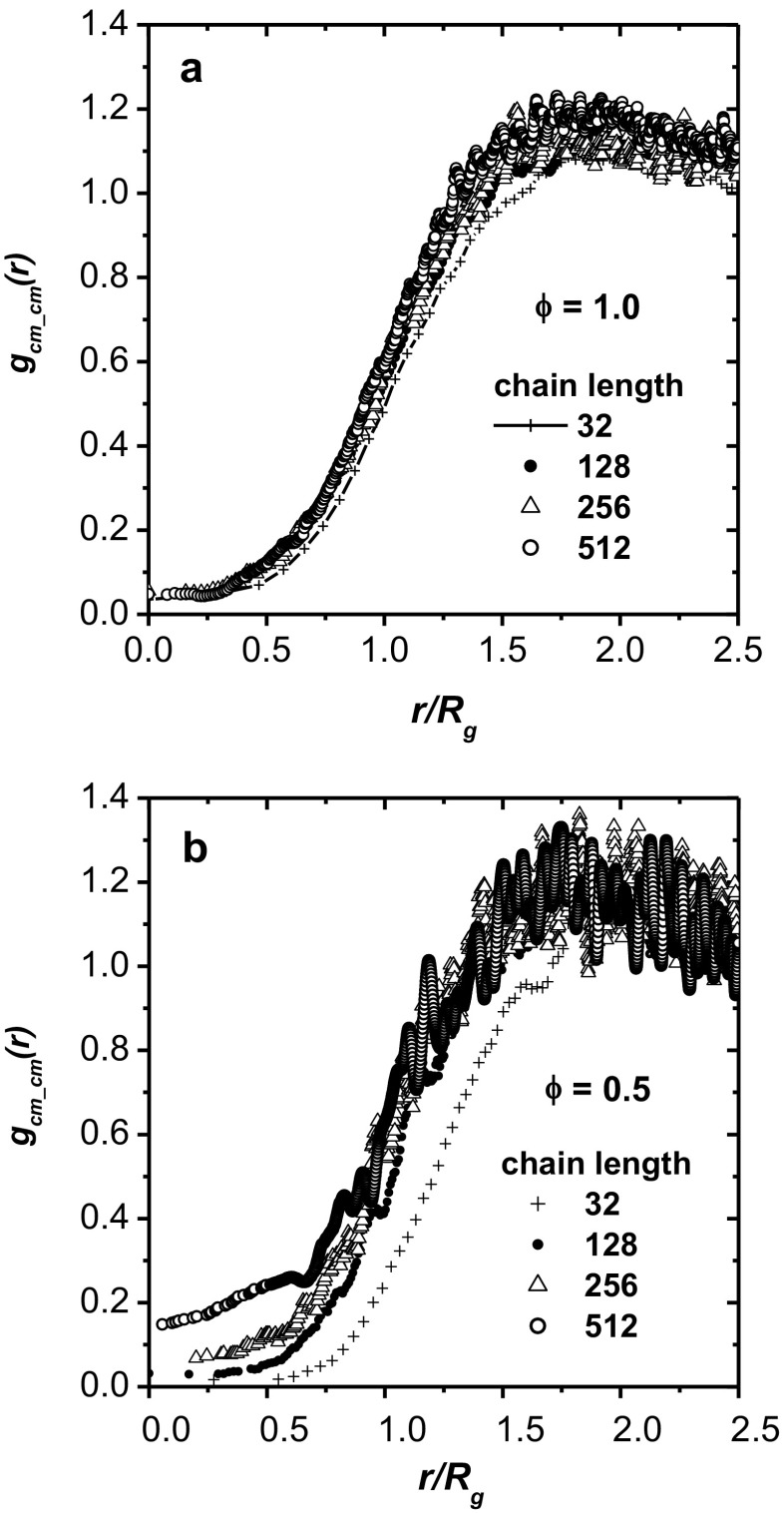
Center-of-mass correlation functions for various chain lengths at *φ* = 0.5 (**a**) and *φ =* 1.0 (**b**)

Two scenarios have been suggested for chain packing in 2D. In the first, it is argued that the chains cannot interpenetrate and therefore the polymer coils must be segregated disks [21]. Such segregation results in a very deep correlation hole because other chains are almost entirely excluded from a region approximately the size of a single chain. On the other hand, in the second scenario (the scaling theory), chains interpenetrate in the semidilute regime [46]. Analysis of the snapshots in Figs. 3 and 5 indicates that interpenetration is a common phenomenon, even in the melt (*φ* = 1). Figure 6 shows the chain center-of-mass correlation function *g*_cm-cm_(*t*) calculated according to Eq. 7 for various chain lengths and plotted in reduced coordinates* r*/*R*_g_ for concentrations *φ* = 0.5 and *φ* = 1, respectively. It is clear that in polymer melts (*φ* = 1), chain interpenetration increases slightly with increasing chain length, but this correlation is weak (Fig. 6a). The values of* g*(*r*)_cm_cm_ for small* r*/*R*_g_ values are also small in all cases. This means that, in a dense system, the exclusion of other chains is strong and the chains are disc-shaped, although the tendency to penetrate other chain coils increases with increasing chain length. Experiments on real 2D systems have revealed that interpenetration occurs in dense polymer systems [18]. For a concentration of *φ* = 0.5, the correlation hole for a chain length *N* of 32 (as a fraction of *R*_g_) is definitely wider, which suggests that short-chain coils exhibit greater separation from each other. Another striking difference is that the values of* g*(*r*) for small* r*/*R*_g_ are much higher for long chains. This may be due to the more oblate shape of long-chain coils and/or the irregular shapes of many of them, which make interpenetration more likely (chain interpenetration is usually considered to be penetration into a circle of a radius *R*_g_; thus, rod-like chains interpenetrate more than disc-like ones).

**Fig. 7:**
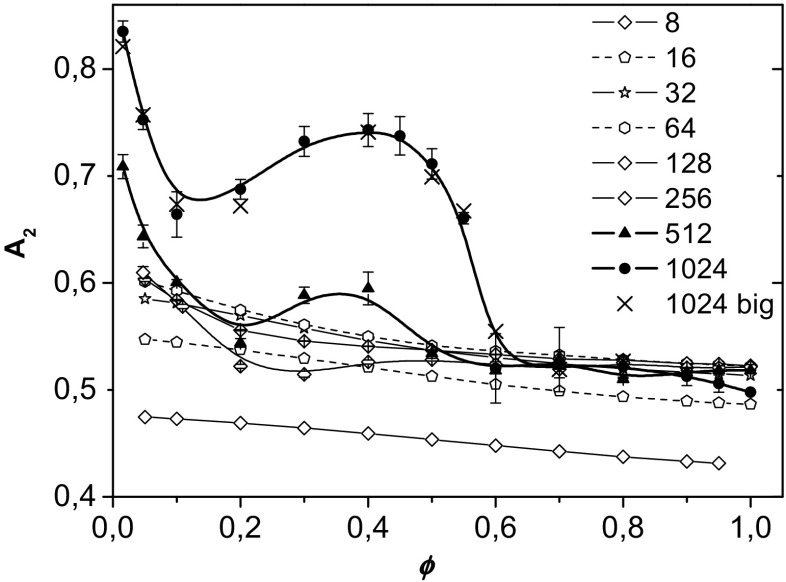
Asphericity parameter *A*_2_ plotted vs. the polymer concentration *φ*. The chain lengths are given in the* inset*. Asphericity values for the longest chains (*N* = 1024) in a large box (512 × 512) are labeled* 1024 big*

It is also interesting to examine how the chain shape changes with increasing polymer concentration for short and long chains. The average chain asphericity *A*_2_ is shown as a function of the polymer concentration *φ* in Fig. 7. For *N* < 256 (thin lines), there is only a small decrease in *A*_2_ with increasing *φ* (ca. 10%, which is similar to the value obtained in off-lattice Monte Carlo simulation studies [1]). Similar behavior is observed for long chains at *φ* values higher than the critical concentration for microphase separation (*φ*_c_ ≅ 0.6). Chains with *N* > 16 exhibit the same asphericity in both a concentrated solution and in the melt, *A*_2_ = 0.52, which is well below the theoretically predicted value of 0.59 [48] or the values of 0.52–0.62 found in previous simulations [1, 31]. At low concentrations, the chain shape for 32 < *N* < 512 is also in reasonable agreement with simulation data (0.63–0.64 [1]) and experimental values (0.61 for real 2D systems [5] and 0.56 for quasi-2D systems [11]). The low values of *A*_2_ for *N* < 32 are probably due to the lattice effect. The values obtained in off-lattice simulations [1] for short and long chains are very similar. The concentration dependence of *A*_2_ for the longest chains is very different. In this case, at low *φ*, the asphericity is very high and decreases sharply with increasing *φ*, but it increases again in the semidilute region. In the concentration range corresponding to phase-separated systems, the asphericity of long chains is high, which is most probably related to anomalous stretching of the chains at the solvent domain boundaries, and it shows considerable scatter. Close to the critical concentration for microphase separation, *A*_2_ drops rapidly before leveling off at higher concentrations. Generally, at low concentrations, the longest chains have a more oblate, rod-like shape than short chains, in contrast to the concentrated regime, where all chains have similar *A*_2_ values and are more disk-like. The simulations performed in the larger box reproduce the nonmonotonic asphericity behavior of longer chains.

### Chain structure

**Fig. 8:**
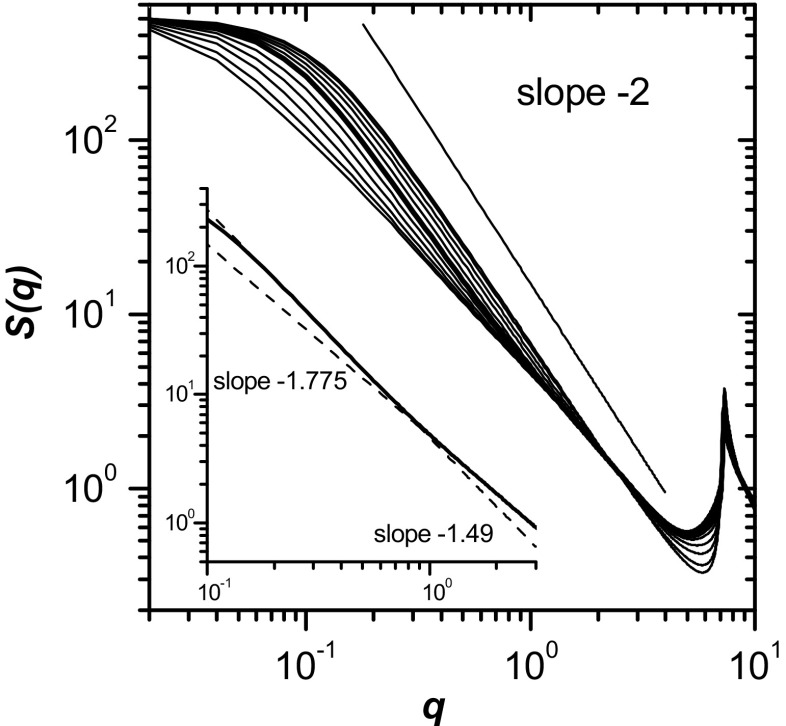
**a** The single-chain structure factor *S*(*q*) for various polymer concentrations (chain length *N* = 512). The *inset *shows an enlargement of the central portion of the plot for *φ* = 0.5 (*thicker line*) to highlight the change in the slope at *q* corresponding to the correlation length

The structural properties of polymer chains can be analyzed using the static structure factor. If we consider a disc of radius *r* around a given bead, and there are *k* beads from the same chain inside that disc, then *γ*(*r*) ∝ *k*/*r* ^2^, where* γ*(*k*) is the intramolecular site–site correlation function defined in Eq. 5. If the mean-square end-to-end distance scales with the number of segments as *R*_ee_^2^ ∼ *k* ^2*ν*^, then $\gamma (r)\propto {r}^{\frac{1}{\nu}-2}$. Taking the Fourier transform and using scaling theory, we obtain the following scaling of the structure factor [1]:

10 $$ S(q)\propto {q}^{-\frac{1}{\nu}}, $$

where *q* is the scattering vector. The situation is more complicated in semidilute solutions when macromolecules behave as ideal chains of “blobs” [21]. In this case, the scaling exponent should be equal to 3/4 within a correlation length *ξ* and equal to 0.5 at a larger scale. Therefore, the slope should change from ca. –4/3 to −2 around* q* = 2π/*ξ* in a plot of log*S*(*q*) vs. log*q*. This effect was indeed observed for simulated chains with lengths of up to *N* = 100^29^. Figure 8 depicts the structure factor of a single chain as a function of the scattering vector *q* for a chain length *N* of 512 at various concentrations. The results shown for high and low polymer concentrations are in agreement with the fractal scattering law (Eq. 10), i.e., the slopes of these curves in Fig. 8 are equal to −4/3 and −2, respectively. Note that the results obtained for a very high concentration of chains (*φ* ∼ 1.0) are in perfect agreement with those attained in a recent experiment on granular chains [13]. Moreover, there do not appear to be any of the deviations that were reportedly obtained in other molecular dynamic simulations [14], where a non-Gaussian chain shape was obtained with slopes of *S*(*q*) approaching 11/4. We also find that the slope at low *q* is indeed higher than that at large *q* for intermediate concentrations (see the inset in Fig. 8). In other words, at large scale, the chains behave as if they are in a dilute solution, whereas they behave as if they are in a dense system at short range.

## Conclusions

We have presented the results of simulations of 2D polymer solutions over a broad range of chain lengths and concentrations, including polymer melts. In contrast to previous simulations performed in this context, solvent molecules were explicitly taken into account. The results obtained for solutions of short chains (*N* < 256) were in good agreement with relevant previous simulations and theory. For the longest chains (512 and 1024 beads), some unexpected behavior in the semidilute and dilute regimes was observed. The radius of gyration, the end-to-end distance, and the chain asphericity all showed a rapid change in concentration dependence at around *φ* = 0.6–0.2, and we propose that this is due to a microphase separation [31]. However, deviations from the models were also observed for the lowest concentrations, below the internal concentration φ*, as the models do not take into account the strong effect of solvent excluded-volume interactions in 2D (solvent incompressibility). The longest chains clearly deviated from the scaling laws: the radius of gyration and the end-to-end distance increased with *N* faster than expected, and their ratio also increased. The chains became more rod-like and their asphericity exceeded 0.8 for *N* = 1024. The results were in agreement with the previous simulations and with theory at high concentrations (*φ* > 0.6), even for the longest chains.

The single-chain scattering structure factor showed changes in the fractal dimension of the macromolecule as a function of concentration. In the semidilute and concentrated regimes, a crossover in the fractal dimension between low and high *q* was observed, in agreement with theory and with the results of previous simulations of shorter chains. This can be used to determine the correlation length. It was found that, for the longest chains, the concentration dependence of the correlation length saturated in the semidilute regime, as expected in the case of phase separation. Simulation snapshots and the center-of-mass correlation function showed an increase in chain interpenetration and a decrease in the correlation hole for long chains (especially at intermediate concentrations). To support the supposition that the observed anomalous behavior was a result of explicit solvent treatments where the solvent molecules could not pass over the chain in 2D, additional simulations were performed in a two-layer simulation box (quasi-2D system) in which the solvent molecules could easily pass over a long chain. The chain size for concentrations above that corresponding to phase separation was found to be the same as the chain size seen in the 2D system, but no anomalous increase was observed at low concentrations for the two-layer system. We can therefore say that long polymer chains behave in a different way in a 2D solution when the solvent is confined in 2D and its excluded volume is taken into account. The presence of an incompressible solvent modifies the probability that the conformation of a chain will change in the vicinity of other chains. This effect is weaker for high concentrations and short chains, so our results agree with the previous findings in such cases.

Two-dimensional systems are also considered a limiting case of confined geometry. The model considered in this work corresponds to polymers intercalated in layered silicates, with solvent molecules only rarely crossing the polymer chains; the model does not correspond to chains adsorbed on a surface, meaning that the solvent molecules can move in 3D space and only the polymer is confined to 2D. It is also worth noting that extrapolations of the results obtained from simulations of short chains must be performed with care—important effects that only become apparent for sufficiently long chains and when considering a broad concentration range can be missed.
